# Authenticity Markers of Aged Red Wines from Aglianico, Uva di Troia, Negroamaro and Primitivo Grapes

**DOI:** 10.3390/foods13121866

**Published:** 2024-06-14

**Authors:** Ilaria Benucci, Claudio Lombardelli, Pasquale Tamborra, Massimo Muganu, Marco Esti

**Affiliations:** 1 Department of Agriculture and Forest Sciences (DAFNE), Tuscia University, Via S. Camillo de Lellis Snc, 01100 Viterbo, Italy; ilaria.be@unitus.it (I.B.); lombardelli@unitus.it (C.L.); muganu@unitus.it (M.M.); 2 Council for Agricultural Research and Economics, Research Center for Viticulture and Enology, CREA-VE Via Casamassima 148, 70010 Turi, Italy; pasquale.tamborra@entecra.it

**Keywords:** red wine, typical Italian grape, glycosidic precursors, shikimic acid, anthocyanins, terpenes, norisoprenoids, hydroxycinnamic acids

## Abstract

The wide ampelographic treasure of Italian wine grape varieties is driving research towards suitable approaches for the varietal authenticity control of wine. In this paper, Aglianico, Negroamaro, Primitivo and Uva di Troia red wines, which were produced experimentally by single-grape winemaking from non-aromatic grapes native to southern Italy, were analyzed with respect to berry markers, namely anthocyanins, hydroxycinnamic acids (HPLC-DAD), shikimic acid (HPLC-UV) and glycosidic aroma precursors (GC-MS). The study confirms that, just as for the berries, useful varietal authenticity markers for red wine, even after aging, turn out to be hydroxycinnamic acids, relative amounts of acylated forms of anthocyanins, and shikimic acid, together with some grape glycosidic precursors from terpenes and C_13_− norisoprenoids. Principal Component Analysis was used as a valuable tool to highlight the results.

## 1. Introduction

Despite the intense research efforts, the availability of suitable approaches to guarantee wine authenticity is still one of the most important issues in the oenological field [1,2,3].

While it has been shown that shikimic acid [4,5], low molecular weight phenolics [6,7,8,9,10,11] and the aroma precursors of berries [12,13,14,15,16] are useful markers in the authentication of grape varieties, it is not clear whether they can also serve in the authentication of the resulting wines, given the dramatic transformations that occur during the process of winemaking, and especially in the case of red wines, during the aging process. Only a few studies have investigated the varietal markers of some typical Italian red wines [17,18,19,20] such as Aglianico, Negroamaro, Uva di Troia, and Primitivo. These are local, non-aromatic red grape vines (*Vitis vinifera* L.), which are widespread in southern Italy, and from which highly regarded red wines can be produced, given their qualities and suitability for aging [21].

In a previous study [22], some useful berry markers for varietal authenticity for these grapes were identified. The most important of these are glycosidic precursors from various terpene families, shikimic acid, acetylated forms of anthocyanins, and hydroxycinnamoyl tartaric acids. The aim of this study was to examine the presence of these same markers in the resulting red wine, in order to see if they could be useful for traceability or verification of authenticity [23,24] of the respective wines, and especially after long periods of aging.

To achieve this purpose, anthocyanins, flavonol glycosides, hydroxycinnamic acids and aroma precursors, as well as shikimic acid and resveratrol, were analyzed in the four varietal red wines at five different stages of aging. These results were then elaborated using Principal Component Analysis.

## 2. Materials and Methods

### 2.1. Wine

The Aglianico, Negroamaro, Uva di Troia, and Primitivo red wines were produced by single-grape pilot-scale winemaking using the homonymous grape at the experimental wine cellar of the Research Center for Viticulture and Enology. The wines were from grapes harvested at commercial maturity (vintage 2012). Bottled samples were aged in the dark at 18 ± 2 °C, and for each wine, 3 bottles were analyzed at each storage time (1, 2, 3, 7 and 11 years).

### 2.2. Extraction and Determination of Phenolics, Glycoconjugates, and Organic Acids

The chemical data set of each varietal wine was obtained applying the following previously developed methods:

- Organic acids were analyzed by HPLC-UV [25];
- Selected flavonoids, phenolic acids and resveratrol were obtained and analyzed (HPLC-DAD) according to Di Stefano [26];
- Terpenic, norisoprenoid and benzenoidic compounds, present as precursors in the glycosylated fraction of wine, were obtained and analyzed (GC-MS) according to Di Stefano [27].
- Chromatic parameters were determined according to official OIV methods.

### 2.3. Chemicals and Reference Compounds

The reagents (Carlo Erba, Milan, Italy) were analytical or HPLC grade, as required. Shikimic acid, caffeic acid, resveratrol, quercitin dihydrate, kaempferol, and myricetin standards in addition to 1-heptanol were from Sigma-Aldrich, Inc. (St. Louis, MO, USA).

### 2.4. Statistical Analysis

All the samples were analyzed in triplicate. Data were analyzed by: (i) two-way analysis of variance (ANOVA) for testing the effect of the single factors (years of aging and variety) and their interaction on the shikimic acid, phenolic and color parameters (Table 1), content of compounds obtained by enzymatic hydrolysis of glycosilated precursors (Table 2) and amount of higher alcohols (Table 3) (*p* < 0.01); and (ii) one-way ANOVA followed by Tukey’s post-hoc test (HSD) (*p* < 0.05, EXCEL^®^ Add-in macro DSAASTAT). Principal Component Analysis (PCA) of data sets was carried out on all variables simultaneously in order to summarize the information on differences between samples. Each variable was standardized for unit variance prior to analysis (Unscrambler, Windows Version 9.6 software package, CAMO A/S, Trondheim, N). Full cross validation was used as the validation criterion.

## 3. Results and Discussion

Table 1 reports the shikimic acid, phenolic compound contents, and color parameters of aged red wines from the four typical Italian varieties. In general, these results confirmed the presence of grape markers in the experimental wines as well negligible changes while aging. Just as was previously found for the grapes [22], Aglianico wines presented significantly higher values of shikimic acid (ranging from 29 to 43 mg/L) than the other varieties irrespective of the aging time. Again reflecting the berry components described in our previous work [22], the wines from the other three varieties showed rather low contents of shikimic acid (max 17.5 mg/L).

Focusing on the percentage of monomeric anthocyanins, it is worth noting the negligible effect of aging on 3-O-glucosidic and acylated forms for all the tested varieties. As was found for the grapes [22], Uva di Troia wines showed a significant higher relative content of acetylated forms (>25%) and a lower content of coumarylated forms with respect to the other wines.

Regarding the amount of hydroxycinnamic acids in wines, resembling those already found in grapes [22], the highest content (171–337 mg/L, related to the highest value of OD 320 nm) was detected in all Negroamaro wines. Meanwhile, the lowest (35–57 mg/L) was found in wines made from Uva di Troia. In addition, the highest amount of resveratrol glucoside previously found in the Negroamaro grape [22], which is attractive for its biological effects, was also maintained in wines even after 7 and 11 years of aging (about 5 mg/L). Finally, in the wines, there were no noteworthy varietal-related differences with respect to flavonols and color-related parameters (total anthocyanins, color intensity, and tonality hue).

In Table 2, the volatiles released by the enzymatic hydrolysis of glycosidic precursors in the four aged red wines are reported. As to the wine levels of glycosidic precursor derivatives of terpenes and C_13_-norisoprenoids, significant differences were found with respect to the variety and years of aging, confirming their potential interest for wine authentication.

In accordance with the results previously described for the grapes [22], Aglianico wines displayed quite high levels of the aglycons released from norisoprenoids, as well as of the main terpene families, while there were no significant effects of aging on these compounds. Irrespective of the aging time, red wines from Negroamaro grapes demonstrated the highest content of linalool and its by-products, namely *trans*- and *cis*-pyran linalool oxide. On the other hand, alfa-terpineol and its derivatives, namely 2-hydroxy-1,8-cineole and p-ment-1-ene-7,8-diol, were very scarce. In addition to the significant higher presence of nerol and geraniol, Uva di Troia red wines were characterized by the highest levels of alfa-terpineol and its derivatives, especially in 7- and 11-year-old wine, while the linalool family compounds were present at low concentrations. As previously found in the grapes [22], all Primitivo red wines displayed a terpene pattern with a prevalence of alfa-terpineol derivatives (particularly of p-ment-1-ene-7,8 diol) during 2 years of aging, and a scarcity of aglycons derived from norisoprenoids.

It is worth noting that, besides nerol, the glycosidic precursor of linalool and alfa terpineol, the two monoterpene representatives of corresponding families, dramatically decreased with aging while their derivatives did not suffer significant variations.

Irrespective of the aging time and variety, no significant differences were revealed in the amount of benzenoids and lipid derivatives, according to that previously found in the four different red grape varieties [22].

The only significant thing to note concerning the content of higher alcohols in the four aged red wines (Table 3) is the significantly higher level of n-propanol and isobutanol in Primitivo than in the other wines.

Five glycosidic precursor ratios of four varietal wines at five different aging times, along with the ratios from the corresponding grapes, are reported in Table 4. Among these ratios that are considered useful for grape and wine characterization [18], only the *trans*-furan linalool oxide vs. *cis*-furan linalool oxide, *trans*-pyran linalool oxide vs. *cis*-pyran linalool oxide, and *trans*-8-hydroxy–linalool vs. *cis*-8-hydroxy-linalool ratios were found to be identical in both wine and the corresponding grape. Additionally, such ratios (bold characters in Table 4) were found to not be affected by the winemaking process or the time of aging, and so they may be considered as useful authenticity markers.

**Figure 1:**
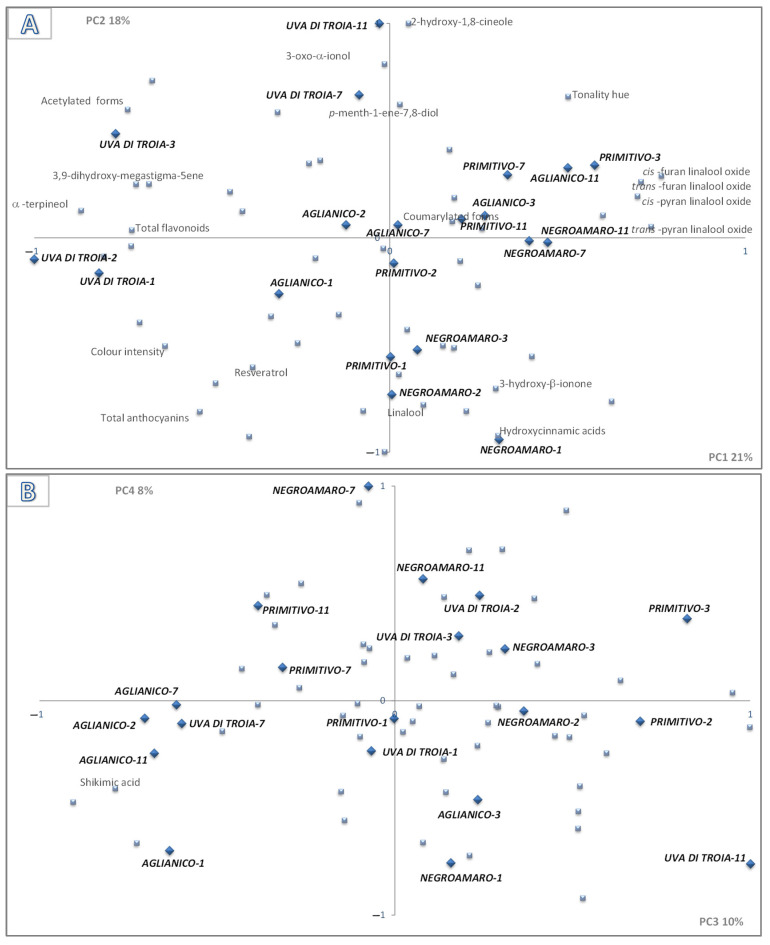
Principal Component Analysis carried out on the complete data matrix reported in Table 1, Table 2 and Table 3. The figures are bi-plots displaying the sample scores (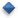) and variable loadings (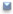) (variable loadings not characteristic are reported as single unlabelled points) in the planes formed by PC1–PC2 (**A**) and PC3–PC4 (**B**).

In order to better interpret the contribution of the 51 analyzed compounds that are reported in Table 1, Table 2 and Table 3 to the characteristic chemical profile of the varietal red wines and to select, among them, the varietal determinants best describing the differences between the resulting wines, a Principal Component Analysis was applied to the data matrix. The matrix contained, as variables, the mean values of 51 compounds and, as samples, the four wine varieties at five aging times. A four-dimensional model, able to account for 57% of the variance, has been judged adequate for reproducing the communalities and the correlations among the variables considered. The two bi-plots derived from the analysis, displaying PC1 vs. PC2 and PC3 vs. PC4, are depicted in Figure 1A and in Figure 1B, respectively. In the figures, all variable loadings, which in our previous work and literature statements were assessed to not be useful for characterizing the varieties considered, are shown as unlabelled points.

The first two dimensions of PCA (39% of variance) discriminate the Uva di Troia from the three other varietal red wines. Uva di Troia samples were both negatively correlated for the first component (1, 2 and 3 years old) and positively correlated for the second component (oldest wines), due to the high presence of alfa-terpineol (74% of variance), acetylated anthocyanins (53%), total flavonoids (52%) and 3,9-dihydroxy-megastigma-5ene (50%), and due to the remarkable amount of 2-hydroxy-1,8-cineole (65%), 3-oxo-alfa-ionol (43%) and *p*-menth-1-ene-7,8-diol (37%), respectively (Figure 1A). Among the three other varietal red wines, Negramaro turned out to be the most different from Uva di Troia wine. These wines (Negroamaro at different aging times), lying on the negative PC2 semi-axis (1, 2 and 3 years old) and on the positive PC1 semi-axis (7 and 11 years old), were characterized by the high presence of hydroxycinnamic acids (64% of variance), linalool (40%), 3-hydroxy-beta-ionone (32%), resveratrol (24%) and by the high content of coumarylated forms of anthocyanins (35%), cis (57%) and trans (49%) -furan linalool oxides, *cis* (48%) and *trans* (53%) -pyran linalool oxides, respectively (Figure 1A).

Primitivo and Aglianico wines occupied an intermediate position, on the plane, between Uva di Troia and Negramaro samples. This distribution of Primitivo and Aglianico was found on the negative PC2 semi-axis (youngest wines) and on the positive PC1 semi-axis (oldest wines). The former were characterized by a high content of hydroxycinnamic acids and *p*-menth-1-ene-7,8-diol, while the latter were characterized by a similar concentration of the derivatives of alpha terpineol and linalool, respectively.

The kind of distribution found for all the wines (oblique with respect to the axes) on the plane of the first two PCs was also affected by the color-related parameter (total anthocyanins, color intensity, tonality hue) position, whose value naturally changes during aging.

The central position, near the origin of axes, that is occupied by the higher alcohols (not labelled in the figure) demonstrates the scant interest of such compounds relative to the authentication of these varietal red wines.

The first two PCs accounted for much of the variables’ variance, but they did not provide a very satisfactory interpretation of the shikimic acid variable (only 3% of variance). However, the third and fourth dimensions of the model (PC3 and PC4) accounted for an additional 18% of variance by explaining the remarkable presence of shikimic acid (44% of variance accounted for on both PCs) in Aglianico samples (Figure 1B).

## 4. Conclusions

The relative amount of glycosidic precursors of linalool and alfa-terpineol derivatives turned out to be, just as in the case of the grapes, helpful authentication markers for the four varietal red wines investigated. Moreover, hydroxycinnamic acids, acylated forms of anthocyanins, and shikimic acid have been confirmed as reliable varietal determinants that are also unaffected by aging in these red wines. In contrast to data observed for the grapes, C_13_-norisoprenoid precursors turned out to provide a meaningful contribution to the differentiation of varietal red wines when aged.

Further useful authenticity markers were found to be the ratios between glycosidic precursors of different forms of linalool oxides, with these being identical in both the grape and the corresponding wine.

Focusing on the effects of aging on composition in the red wines from the four investigated varieties, Aglianico demonstrated, just as for the grapes, a remarkably high presence of shikimic acid that was unaffected by aging as well as the similar concentration of the derivatives of alpha terpineol and linalool, respectively. Negroamaro wines were characterized, even when aged, by their high content, just as in the grape, of hydroxycinnamic acids, resveratrol glucoside and linalool or its derivatives (oldest wines). Uva di Troia red wines maintained high grape-related contents of acetylated anthocyanins and alfa-terpineol family derivatives. Also reflecting the profile of the grapes, Primitivo wines showed remarkably stable contents of hydroxycinnamic acids.

Overall, the PCA approach has proven to be very useful for the authentication of these varietal wines throughout their lifetime.

## Figures and Tables

**Table 1:** Shikimic acid, phenolic and color parameters (mean ± SD) ^§^ of aged red wines. Values with different lowercase letters (^a–e^) differ significantly (Tukey’s test, *p* < 0.05) according to years of aging. Values with different capital letters (^A–D^) differ significantly (Tukey’s test, *p* < 0.05) according to variety.

	**Aglianico**					**Negroamaro**				
	**1** **Year**	**2** **Years**	**3** **Years**	**7** **Years**	**11** **Years**	**1** **Year**	**2** **Years**	**3** **Years**	**7** **Years**	**11** **Years**
Shikimic acid (mg/L)	43.1 ± 2.1 ^a, A^	32.3 ± 1.4 ^c, A^	36.3 ± 1.8 ^b, A^	31.8 ± 2.1 ^c, A^	29.1 ± 2.0 ^c, A^	10.0 ± 0.6 ^c, B^	5.2 ± 0.7 ^d, C^	15.0 ± 1.3 ^ab, B^	14.0 ± 1.4 ^b, B^	17.5 ± 1.4 ^a, B^
3-O-glucosidic forms of anthocyanins (%)	75.1 ± 5.9 ^ab, AB^	62.7 ± 4.9 ^b, B^	74.2 ± 6.2 ^ab, A^	77.5 ± 4.4 ^a, A^	61.9 ± 2.5 ^b, A^	74.3 ± 6.5 ^ab, AB^	61.0 ± 4.7 ^bc, B^	80.5 ± 6.3 ^a, A^	54.2 ± 6.1 ^c, B^	61.4 ± 3.9 ^c, A^
Acetylated forms of anthocyanins (%)	11.0 ± 1.7 ^a, B^	3.1 ± 0.8 ^c, B^	6.9 ± 0.8 ^b, BC^	6.3 ± 1.2 b ^c, B^	7.4 ± 1.3 ^b, B^	4.6 ± 0.7 ^c, C^	9.8 ± 0.9 ^a, B^	3.5 ± 0.5 ^c, C^	2.9 ± 0.8 ^c, B^	7.0 ± 0.3 ^b, B^
Coumarylated forms of anthocyanins (%)	13.9 ± 2.4 ^b, B^	34.2 ± 3.2 ^a, A^	18.9 ± 1.0 ^b, B^	16.2 ± 2.7 ^b, B^	30.7 ± 3.3 ^a, A^	21.1 ± 2.5 ^cd, A^	29.2 ± 1.9 ^bc, A^	16.0 ± 1.4 ^d, B^	42.9 ± 5.8 ^a, A^	31.6 ± 4.4 ^b, A^
Hydroxycinnamic acids (mg/L)	176.0 ± 12.5 ^a, B^	72.9 ± 5.1 ^d, C^	91.6 ± 5.4 ^c, C^	108.8 ± 8.6 ^b, B^	50.1 ± 4.7 ^e, C^	326.8 ± 29.1 ^a, A^	163.2 ± 21.5 ^b, A^	337.7 ± 30.3 ^a, A^	171.0 ± 5.3 ^c, A^	146.5 ± 11.3 ^b, A^
Flavonols (mg/L)	10.9 ± 1.1 ^a, A^	7.5 ± 0.4 ^b, B^	4.7 ± 0.5 ^c, B^	1.6 ± 0.9 ^d, B^	0.7 ± 0.4 ^d, B^	0.7 ± 0.3 ^d, C^	7.1 ± 0.5 ^b, B^	9.0 ± 0.8 ^a, A^	2.7 ± 0.4 ^c, A^	1.0 ± 0.2 ^d, B^
Resveratrol (mg/L)	1.6 ± 0.7 ^a, C^	1.2 ± 0.4 ^b, C^	0.2 ± 0.1 ^c, C^	0.3 ± 0.3 ^c, BC^	0.2 ± 0.2 ^c, C^	0.9 ± 0.6 ^d, D^	6.7 ± 0.8 ^a, A^	3.9 ± 0.4 ^c, A^	4.9 ± 0.5 ^b, A^	4.8 ± 0.6 ^bc, A^
O.D. 280 nm	60.4 ± 0.5 ^ab, A^	59.3 ± 0.7 ^b, B^	33.8 ± 0.4 ^d, C^	62.4 ± 0.5 ^a, A^	48.5 ± 0.7 ^c, C^	44.4 ± 0.8 ^d, B^	51.3 ± 1.1 ^c, C^	56.1 ± 1.0 ^b, B^	40.8 ± 0.9 ^e, C^	59.4 ± 0.6 ^a, A^
O.D. 320 nm	21.7 ± 0.3 ^bc, B^	25.9 ± 0.6 ^a, B^	19.9 ± 0.5 ^c, B^	26.3 ± 0.5 ^a, A^	21.7 ± 0.8 ^b, B^	29.3 ± 0.4 ^b, A^	32.1 ± 0.5 ^a, A^	31.0 ± 0.9 ^ab, A^	19.5 ± 0.3 ^d, AB^	26.0 ± 0.4 ^c, A^
O.D. 280 vs. O.D.320	2.8 ± 0.2 ^a, A^	2.3 ± 0.1 ^b, B^	1.7 ± 0.2 ^c, B^	2.4 ± 0.1 ^b, B^	2.2 ± 0.1 ^b, B^	1.5 ± 0.1 ^c, B^	1.6 ± 0.1 ^bc, C^	1.8 ± 0.1 ^b, B^	2.1 ± 0.1 ^a, BC^	2.3 ± 0.1 ^a, B^
λ max	277.5 ± 0.5 ^BC^	277.5 ± 0.5 ^AB^	279.0 ± 0.5 ^A^	277.5 ± 0.5 ^A^	277.5 ± 0.5 ^A^	280.0 ± 0.5 ^a, A^	280.0 ± 0.5 ^a, A^	279.5 ± 0.5 ^a, A^	277.5 ± 0.5 ^b, A^	278.5 ± 0.5 ^ab, A^
Total flavonoids (mg/L)	2307 ± 15 ^a, A^	1934 ± 31 ^b, A^	948 ± 25 ^d, C^	1961 ± 21 ^b, A^	1236 ± 45 ^c, C^	1051 ± 43 ^d, C^	1360 ± 62 ^c, C^	1813 ± 79 ^a, B^	989 ± 43 ^e, C^	1483 ± 52 ^b, B^
Total anthocyanins (mg/L)	276 ± 5 ^a, B^	156 ± 11 ^b, C^	56 ± 7 ^d, C^	111 ± 10 ^c, A^	63 ± 5 ^d, AB^	227 ± 11 ^a, C^	246 ± 12 ^a, A^	150 ± 10 ^b, A^	75 ± 8 ^c, B^	62 ± 4 ^c, AB^
Color intensity	11.8 ± 0.3 ^a, A^	9.8 ± 0.2 ^b, A^	4.4 ± 0.2 ^d, C^	9.4 ± 0.4 ^b, A^	6.7 ± 0.2 ^c, B^	8.4 ± 0.3 ^b, B^	9.8 ± 0.5 ^a, A^	5.5 ± 0.3 ^d, B^	5.9 ± 0.4 ^d, BC^	7.4 ± 0.2 ^c, A^
Tonality hue	0.6 ± 0.1 ^d, B^	0.8 ± 0.1 ^c, D^	0.9 ± 0.1 ^b, A^	0.9 ± 0.1 ^b, C^	1.2 ± 0.1 ^a, A^	0.8 ± 0.1 ^c, A^	1.1 ± 0.1 ^b, B^	1.0 ± 0.1 ^b, A^	1.0 ± 0.0 ^b, BC^	1.2 ± 0.1 ^a, A^
	**Uva di troia**					**Primitivo**				
	**1** **year**	**2** **years**	**3** **years**	**7** **years**	**11** **years**	**1** **year**	**2** **years**	**3** **years**	**7** **years**	**11** **years**
Shikimic acid (mg/L)	6.7 ± 0.6 ^b, C^	7.2 ± 1.0 ^b, C^	11.5 ± 1.2 ^a, C^	11.1 ± 1.3 ^a, C^	8.7 ± 0.8 ^b, C^	11.0 ± 0.9 ^b, B^	10.0 ± 0.3 ^b, B^	9.0 ± 0.7 ^b, C^	16.0 ± 1.2 ^a, B^	16.1 ± 1.1 ^a, B^
3-O-glucoside forms of anthocyanins (%)	64.9 ± 6.6 ^B^	53.2 ± 4.9 ^B^	56.3 ± 3.9, ^B^	56.9 ± 4.9 ^B^	60.0 ± 6.9 ^A^	82.6 ± 7.7 ^a, A^	86.2 ± 2.2 ^a, A^	54.3 ± 5.9 ^b, B^	47.6 ± 4.9 ^b, B^	55.4 ± 6.3 ^b, A^
Acetylated forms (%)	26.6 ± 3.8 ^A^	29.8 ± 5.0 ^A^	33.3 ± 3.4 ^A^	27.6 ± 3.3 ^A^	25.7 ± 3.2 ^A^	8.3 ± 0.9 ^b, BC^	7.9 ± 3.8 ^bc, B^	13.3 ± 3.5 ^a, B^	6.9 ± 1.7 ^c, B^	11.0 ± 1.4 ^a, B^
Coumarylated forms (%)	8.5 ± 1.5 ^cd, C^	16.9 ± 1.7 ^ab, B^	10.4 ± 0.9 ^bc, B^	15.5 ± 1.7 ^a, B^	14.3 ± 1.4 ^ab, B^	9.2 ± 1.9 ^c, B^	5.9 ± 0.3 ^d, C^	32.4 ± 6.1 ^b, A^	45.5 ± 3.9 ^a, A^	33.6 ± 3.2 ^b, A^
Hydroxycinnamic acids (mg/L)	57.1 ± 3.9 ^a, D^	35.6 ± 3.4 ^c, D^	47.1 ± 3.4 ^b, D^	42.0 ± 3.4 ^bc, D^	34.9 ± 3.4 ^c, C^	146.5 ± 12.3 ^ab, A^	160.9 ± 11.8 ^a, A^	126.4 ± 9.3 ^b, AB^	89.6 ± 5.8 ^c, C^	123 ± 10.6 ^b, B^
Flavonols (mg/L)	12.2 ± 1.3 ^b, A^	22.2 ± 1.4 ^a, A^	2.4 ± 0.9 ^c, C^	1.4 ± 0.7 ^c, B^	1.1 ± 0.4 ^c, B^	6.0 ± 0.9 ^b, B^	2.3 ± 0.4 ^c, C^	0.9 ± 0.6 ^d, D^	1.3 ± 0.3 ^cd, B^	8.6 ± 0.6 ^a, A^
Resveratrol (mg/L)	4.9 ± 0.2 ^b, A^	7.4 ± 0.7 ^a, A^	0.8 ± 0.3 ^c, B^	0.7 ± 0.9 ^c, B^	0.3 ± 0.2 ^c, C^	2.2 ± 0.2 ^b, B^	3.0 ± 0.4 ^a, B^	0.1 ± 0.0 ^d, A^	0.1 ± 0.0 ^d, C^	1.4 ± 0.2 ^c, B^
O.D. 280 nm	62.3 ± 0.7 ^c, A^	68.6 ± 0.4 ^b, A^	79.6 ± 0.5 ^a, A^	52.6 ± 0.3 ^d, B^	39.2 ± 0.6 ^e, D^	36.5 ± 0.2 ^b, C^	52.5 ± 0.5 ^a, C^	32.5 ± 0.7 ^b, C^	37.0 ± 0.4 ^b, D^	52.5 ± 0.8 ^a, B^
O.D. 320 nm	22.8 ± 0.5 ^ab, B^	24.5 ± 0.3 ^a, B^	21.3 ± 0.2 ^b, B^	16.5 ± 0.3 ^c, C^	13.3 ± 0.5 ^d, C^	21.3 ± 0.4 ^b, B^	25.7 ± 0.5 ^a, B^	21.0 ± 0.5 ^b, B^	18.7 ± 0.9 ^c, B^	22.0 ± 0.3 ^b, B^
O.D. 280 vs. O.D. 320	2.7 ± 0.1 ^c, A^	2.8 ± 0.1 ^c, A^	3.7 ± 0.2 ^a, A^	3.2 ± 0.2 ^b, A^	2.9 ± 0.1 ^bc, A^	1.7 ± 0.1 ^cd, B^	2.0 ± 0.1 ^b, B^	1.5 ± 0.1 ^d, B^	2.0 ± 0.1 ^bc, C^	2.4 ± 0.2 ^a, B^
λ max	275.5 ± 0.1 ^C^	276.0 ± 0.1 ^B^	276.0 ± 0.1 ^B^	276.0 ± 0.1 ^A^	275.0 ± 0.1 ^B^	278.5 ± 0.1 ^AB^	278.5 ± 0.1 ^AB^	280.5 ± 0.1 ^A^	277.5 ± 0.1 ^A^	277.5 ± 0.1 ^A^
Total flavonoids (mg/L)	1875 ± 75 ^b, B^	1920 ± 47 ^b, A^	2802 ± 98 ^a, A^	1730 ± 75 ^c, B^	989 ± 57 ^d, D^	1009 ± 69 ^c, D^	1434 ± 53 ^a, B^	906 ± 38 ^b, D^	865 ± 84 ^b, D^	1566 ± 98 ^a, A^
Total anthocyanins (mg/L)	395 ± 21 ^a, A^	180 ± 19 ^b, B^	78 ± 11 ^c, B^	50 ± 5 ^d, C^	46 ± 5 ^d, B^	205 ± 12 ^a, D^	96 ± 10 ^b, D^	42 ± 7 ^d, C^	74 ± 5 ^c, B^	65 ± 9 ^c, A^
Color intensity	11.5 ± 0.1 ^a, A^	9.4 ± 0.1 ^b, A^	8.7 ± 0.1 ^b, A^	5.8 ± 0.1 ^c, C^	4.9 ± 0.1 ^c, C^	6.7 ± 0.1 ^b, C^	7.8 ± 0.1 ^a, B^	4.0 ± 0.1 ^c, C^	6.6 ± 0.1 ^d, B^	6.7 ± 0.1 ^b, B^
Tonality hue	0.6 ± 0.1 ^c, B^	0.9 ± 0.1 ^b, C^	1.1 ± 0.8 ^a, A^	1.2 ± 0.1 ^a, A^	1.2 ± 0.1 ^a, A^	0.8 ± 0.1 ^b, A^	1.1 ± 0.1 ^a, A^	1.2 ± 0.1 ^a, A^	1.1 ± 0.1 ^a, AB^	1.2 ± 0.9 ^a, A^

^§^ Mean ± standard deviation of three replications of the same sample.

**Table 2:** Content (mean ± SD) ^§^ of compounds (μg/L of 1-heptanol) obtained by enzymatic hydrolysis of glycosilated precursors of aged red wines. Values with different lowercase letters (^a–d^) differ significantly (Tukey’s test, *p* < 0.05) according to years of aging. Values with different capital letters (^A–D^) differ significantly (Tukey’s test, *p* < 0.05) according to variety.

	**Aglianico**					**Negroamaro**				
	**1** **Year**	**2** **Years**	**3** **Years**	**7** **Years**	**11** **Years**	**1** **Year**	**2** **Years**	**3** **Years**	**7** **Years**	**11** **Years**
Terpenes										
*trans*-furan linalool oxide	12 ± 2 ^c,B^	15 ± 3 ^c, B^	38 ± 8 ^b, B^	15 ± 3 ^c, B^	56 ± 7 ^a, A^	46 ± 15 ^A^	48 ± 8 ^A^	36 ± 7 ^B^	37 ± 11 ^A^	53 ± 9 ^A^
*cis*-furan linalool oxide	13 ± 1 ^c, A^	15 ± 3 ^c, A^	27 ± 4 ^b, A^	13 ± 1 ^c, B^	51 ± 6 ^a, A^	13 ± 1 ^cd, A^	19 ± 2 ^bc, A^	7 ± 1 ^d, B^	23 ± 5 ^b, A^	39 ± 7 ^a, A^
linalool	8 ± 1 ^B^	n.d.	n.d.	n.d.	n.d.	38 ± 7 ^a, A^	12 ± 2 ^b^	n.d.	n.d.	n.d.
*trans*-pyran linalool oxide	14 ± 4 ^c, B^	18 ± 7 ^bc, B^	26 ± 6 ^ab, B^	10 ± 2 ^c, B^	35 ± 9 ^a, B^	31 ± 6 ^b, A^	27 ± 7 ^b, A^	26 ± 2 ^b, B^	27 ± 8 ^b, A^	56 ± 8 ^a, A^
*cis*-pyran linalool oxide	6 ± 1 b ^c, A^	7 ± 2 ^bc, B^	10 ± 3 ^b, B^	5 ± 1 ^c^	16 ± 4 ^a, A^	4 ± 1 ^b, B^	5 ± 1 ^b, B^	4 ± 2 ^b, C^	6 ± 3 ^b^	18 ± 5 ^a, A^
*trans*-8-hydroxy-linalool	69 ± 8 ^a, A^	52 ± 9 ^b, A^	17 ± 5 ^c, A^	28 ± 3 ^c, B^	50 ± 10 ^b, A^	23 ± 7 ^a, B^	24 ± 4 ^a, B^	3 ± 2 ^c, B^	5 ± 2 ^c, C^	11 ± 3 ^b, C^
*cis*-8-hydroxy-linalool	125 ± 19 ^a, A^	115 ± 11 ^ab, A^	39 ± 4 ^d, A^	61 ± 7 ^c, A^	103 ± 7 ^b, A^	149 ± 10 ^a, A^	90 ± 8 ^b, B^	21 ± 3 ^c, B^	28 ± 3 ^c, B^	22 ± 2 ^c, C^
nerol	16 ± 3 ^B^	n.d.	n.d.	n.d.	n.d.	12 ± 5 ^B^	n.d.	n.d.	n.d.	n.d.
geraniol	46 ± 8 ^a, B^	31 ± 9 ^b, B^	18 ± 4 ^c, C^	4 ± 2 ^d, C^	19 ± 5 ^c, B^	26 ± 11 ^bc, B^	58 ± 17 ^a, B^	31 ± 6 ^b, B^	13 ± 6 ^cd, B^	4 ± 2 ^d, C^
geranic acid	26 ± 5 ^a, C^	23 ± 7 ^a, C^	29 ± 3 ^a, B^	2 ± 1 ^b, C^	5 ± 1 ^b, B^	34 ± 8 ^c, C^	71 ± 7 ^a, B^	52 ± 2 ^b, A^	8 ± 4 ^d, B^	6 ± 4 ^d, B^
α-terpineol	18 ± 7 ^a, B^	12 ± 4 ^a, B^	5 ± 1 ^b, B^	1 ± 1 ^b, C^	2 ± 1 ^b, B^	14 ± 3 ^a, B^	3 ± 1 ^b, C^	3 ± 1 ^b, B^	4 ± 1 ^b, B^	2 ± 1 ^b, B^
2-hydroxy-1,8-cineole	17 ± 5 ^ab, A^	18 ± 7 ^b, AB^	13 ± 5 ^b, AB^	23 ± 9 ^ab, B^	31 ± 12 ^a, B^	2 ± 1 ^c, C^	9 ± 2 ^bc, B^	2 ± 1 ^c, C^	18 ± 8 ^a, B^	12 ± 6 ^ab, C^
*p*-menth-1-ene-7,8-diol	72 ± 15 ^b, A^	52 ± 11 ^b, B^	54 ± 6 ^b, A^	181 ± 18 ^a, A^	51 ± 5 ^b, C^	12 ± 7 ^c, B^	10 ± 8 ^c, C^	3 ± 1 ^c, C^	28 ± 6 ^b, B^	46 ± 13 ^a, C^
C_13_-Norisoprenoids										
3-hydroxy-β-damascone	84 ± 7 ^a, A^	103 ± 17 ^a, B^	14 ± 6 ^c, C^	36 ± 10 ^b, C^	37 ± 12 ^b, C^	70 ± 14 ^c, A^	58 ± 16 ^c, C^	15 ± 7 ^d, C^	203 ± 20 ^a, A^	103 ± 13 ^b, A^
3-oxo-α-ionol	213 ± 17 ^a, A^	211 ± 15 ^a, A^	151 ± 11 ^b, B^	29 ± 2 ^d, D^	117 ± 9 ^c, B^	127 ± 12 ^a, B^	134 ± 21 ^a, B^	23 ± 9 ^b, C^	143 ± 13 ^a, C^	128 ± 25 ^a, B^
3,9-dihydroxy-megastigma-5 ene	41 ± 6 ^c, B^	82 ± 9 ^a, B^	53 ± 9 ^bc, B^	16 ± 3 ^d, B^	56 ± 12 ^b, A^	5 ± 3 ^b, C^	56 ± 8 ^a, C^	11 ± 8 ^b, C^	9 ± 6 ^b, C^	12 ± 8 ^b, B^
3-hydroxy-β-ionone	112 ± 24 ^B^	n.d.	n.d.	n.d.	n.d.	194 ± 18 ^a, A^	n.d.	96 ± 5 ^c^	116 ± 8 ^b, A^	56 ± 4 ^d^
Benzenoids										
benzoic aldehyde	21 ± 5 ^a^	7 ± 2 ^c, B^	3 ± 1 ^c, C^	6 ± 1 ^c^	14 ± 4 ^b, A^	29 ± 6 ^a^	22 ± 8 ^ab, A^	5 ± 1 ^d, B^	10 ± 6 ^cd^	18 ± 3 ^bc, A^
methyl salicylate	13 ± 1 ^ab, B^	16 ± 3 ^a, A^	10 ± 3 ^b, B^	5 ± 2 ^c^	2 ± 1 ^c, C^	9 ± 2 ^b, C^	16 ± 4 ^a, A^	2 ± 1 ^c, C^	2 ± 1 ^c^	6 ± 3 ^bc, AB^
benzyl alcohol	1176 ± 77 ^a, B^	435 ± 32 ^c, C^	807 ± 61 ^b, B^	288 ± 31 ^d, C^	404 ± 63 ^c, C^	1525 ± 142 ^a, A^	1011 ± 56 ^b, A^	387 ± 33 ^c, C^	422 ± 69 ^c, B^	481 ± 99 ^c, B^
2-phenyl ethanol	568 ± 73 ^a, A^	450 ± 62 ^bc, B^	637 ± 49 ^a, B^	419 ± 53 ^c, B^	551 ± 67 ^ab, B^	472 ± 91 ^B^	491 ± 56 ^B^	585 ± 43 ^B^	579 ± 86 ^A^	534 ± 37 ^B^
eugenol	3 ± 4^c, B^	5 ± 3^bc, C^	21 ± 7^a, C^	5 ± 2 ^bc, C^	11 ± 4 ^b, D^	79 ± 6 ^b, A^	86 ± 14 ^b, A^	119 ± 16 ^a, A^	44 ± 6 ^c, A^	67 ± 9 ^b, A^
vanillin	24 ± 7^b, B^	4 ± 3^cd, B^	56 ± 8^a, A^	13 ± 7^c, B^	2 ± 1 ^d, B^	52 ± 6 ^b, A^	85 ± 4 ^a, A^	11 ± 6 ^d, B^	39 ± 7 ^c, A^	12 ± 6 ^d, A^
methyl vanillate	56 ± 6 ^a, C^	43 ± 11 ^b, C^	59 ± 3 ^a, B^	16 ± 7 ^c, B^	5 ± 2 ^c, C^	149 ± 22 ^b, AB^	204 ± 35 ^a, A^	89 ± 11 ^c, A^	34 ± 7 ^d, A^	11 ± 4 ^d, B^
acetovanillone	12 ± 4 ^ab, B^	19 ± 5 ^a, B^	9 ± 4 ^b^	12 ± 4 ^ab, B^	10 ± 3 ^b, B^	38 ± 3 ^a, A^	32 ± 9 ^ab, A^	12 ± 4 ^b^	41 ± 14 ^a, A^	52 ± 24 ^a, A^
zingerone	6 ± 3 b^c, D^	13 ± 3 ^b, C^	84 ± 12 ^a, A^	6 ± 2 b^c, D^	2 ± 1 ^c, C^	93 ± 16 ^a, A^	55 ± 8 ^b, B^	79 ± 9 ^a, A^	39 ± 6 ^b, A^	51 ± 6 ^b, A^
homovanillic alcohol	43 ± 13 ^bc, B^	132 ± 21 ^a, A^	58 ± 15 b ^c, B^	35 ± 8 ^c, B^	68 ± 16 ^b, B^	72 ± 11 ^b, A^	32 ± 6 ^c, C^	37 ± 6 ^c, C^	117 ± 19 ^a, A^	57 ± 7 ^b, B^
methyl syringate	562 ± 48 ^a, A^	156 ± 24 ^c, C^	287 ± 36 ^b, AB^	59 ± 7 ^d, A^	31 ± 7 ^d, B^	13 ± 8 ^c, C^	784 ± 155 ^a, A^	322 ± 43 ^b, A^	38 ± 6 ^c, B^	4 ± 2 ^c, C^
dihydroconiferyl alcohol	13 ± 8 ^a^	5 ± 3 ^ab^	3 ± 2 ^b, B^	4 ± 3 ^c, C^	8 ± 4 ^ab, AB^	8 ± 2 ^b^	9 ± 4 ^b^	11 ± 4 ^b, A^	62 ± 21 ^a, A^	18 ± 7 ^b, A^
Lipid derivatives										
hexanol	156 ± 18 ^a, A^	163 ± 8 ^a, C^	163 ± 19 ^a, B^	91 ± 16 ^b, A^	84 ± 7 ^b, C^	109 ± 12 ^c, B^	145 ± 21 ^ab, C^	129 ± 9 ^bc, C^	65 ± 4 ^d, B^	166 ± 11 ^a, A^
*cis*-3-hexenol	29 ± 8 ^a, A^	26 ± 3 ^a^	33 ± 3 ^a, B^	27 ± 9 ^a, A^	14 ± 4 ^b, B^	17 ± 3 ^ab, B^	18 ± 5 ^ab^	26 ± 6 ^a, C^	11 ± 3 ^b, C^	23 ± 8 ^a, B^
*trans*-2-hexenol	48 ± 4 ^a, A^	33 ± 8 ^b, AB^	47 ± 8 ^a, A^	15 ± 2 ^c, B^	16 ± 7 ^c, C^	17 ± 7 ^bc, C^	32 ± 12 ^a^	22 ± 6 ^abc, B^	9 ± 3 ^c, B^	29 ± 8 ^ab, B^
	**Uva di troia**					**Primitivo**				
	**1** **year**	**2** **years**	**3** **years**	**7** **years**	**11** **years**	**1** **year**	**2** **years**	**3** **years**	**7** **years**	**11** **years**
Terpenes										
*trans*-furan linalool oxide	5 ± 2 ^c, C^	7 ± 3 ^c, C^	15 ± 6 ^b, C^	19 ± 3 ^b, B^	68 ± 5 ^a, A^	12 ± 2 ^c, B^	19 ± 3 ^c, B^	87 ± 7 ^a, A^	31 ± 5 ^b, A^	30 ± 8 ^b, B^
*cis*-furan linalool oxide	2 ± 1 ^c, C^	2 ± 1 ^c, B^	1 ± 1 ^c, C^	9 ± 3 ^b, C^	21 ± 5 ^a, C^	6 ± 2 ^c, B^	14 ± 3 ^bc, A^	28 ± 7 ^ab, A^	19 ± 4 ^bc, A^	21 ± 5 ^ab, C^
linalool	3 ± 2 ^C^	n.d.	n.d.	n.d.	n.d.	3 ± 1 ^C^	n.d.	n.d.	n.d.	n.d.
*trans*-pyran linalool oxide	8 ± 3 ^b, C^	9 ± 2 ^b, C^	19 ± 5 ^a, B^	23 ± 5 ^a, AB^	18 ± 6 ^a, C^	17 ± 3 ^c, B^	20 ± 4 ^bc, B^	55 ± 8 ^a, A^	23 ± 5 ^bc, AB^	29 ± 7 ^b, B^
*cis*-pyran linalool oxide	2 ± 1 ^C^	2 ± 1 ^C^	2 ± 1 ^C^	3 ± 1	2 ± 1 ^C^	2 ± 1 ^c, C^	9 ± 2 ^b, A^	17 ± 6 ^ab, A^	9 ± 4 ^b^	11 ± 2 ^ab, B^
*trans*-8-hydroxy-linalool	3 ± 2 ^c, D^	7 ± 2 b ^c, C^	4 ± 1 ^c, B^	54 ± 8 ^a, A^	13 ± 3 ^b, C^	11 ± 3 ^d, C^	23 ± 3 ^c, B^	12 ± 4 ^d, A^	50 ± 9 ^a, A^	39 ± 5 ^b, B^
*cis*-8-hydroxy-linalool	33 ± 9 ^b, B^	29 ± 5 ^b, C^	18 ± 8 ^b, B^	59 ± 13 ^a, A^	18 ± 10 ^b, C^	45 ± 12 ^bc, B^	40 ± 6 ^c, C^	16 ± 8 ^d, B^	59 ± 4 ^b, A^	83 ± 6 ^a, B^
nerol	89 ± 4 ^b, A^	166 ± 9 ^a^	44 ± 6 ^c^	n.d.	n.d.	n.d.	n.d.	n.d.	n.d.	n.d.
geraniol	152 ± 20 ^c, A^	286 ± 43 ^a, A^	56 ± 7 ^d, A^	40 ± 6 ^d, A^	216 ± 8 ^b, A^	36 ± 17 ^a, B^	31 ± 9 ^ab, B^	36 ± 11 ^a, B^	39 ± 7 ^a, A^	12 ± 6 ^b, B^
geranic acid	67 ± 9 ^b, A^	89 ± 5 ^a, A^	18 ± 6 ^c, B^	n.d.	n.d.	41 ± 7 ^a, B^	n.d.	42 ± 11 ^a, A^	13 ± 7 ^b, A^	18 ± 3 ^b, A^
α-terpineol	37 ± 12 ^a, A^	33 ± 9 ^a, A^	44 ± 12 ^a, A^	10 ± 6 ^b, B^	12 ± 4 ^b, A^	7 ± 2 ^b, C^	4 ± 1 ^bc, C^	4 ± 1 ^bc, B^	11 ± 3 ^a, AB^	2 ± 1 ^c, B^
2-hydroxy-1,8-cineole	17 ± 4 ^b, A^	18 ± 6 ^b, AB^	26 ± 11 ^b, A^	68 ± 15 ^a, A^	64 ± 13 ^a, A^	10 ± 3 ^b, B^	23 ± 7 ^b, A^	27 ± 9 ^b, A^	67 ± 21 ^a, A^	25 ± 15 ^b, B^
*p*-menth-1-ene-7,8-diol	13 ± 8 ^b, B^	8 ± 3 ^b, C^	12 ± 3 ^b, BC^	25 ± 3 ^b, B^	805 ± 99 ^a, A^	94 ± 21 ^b, A^	153 ± 8 ^a, A^	8 ± 6 ^d, C^	53 ± 31 ^c, B^	99 ± 12 ^b, B^
C_13_-Norisoprenoids										
3-hydroxy-β-damascone	68 ± 7 ^a, B^	68 ± 7 ^a, C^	37 ± 12 ^c, B^	51 ± 7 ^b, B^	56 ± 4 ^ab, C^	86 ± 6 ^c, A^	179 ± 14 ^a, A^	113 ± 10 ^b, A^	53 ± 8 ^d, B^	99 ± 13 ^bc, B^
3-oxo-α-ionol	114 ± 16 ^c, B^	66 ± 8 ^c, C^	261 ± 13 ^b, A^	224 ± 41 ^b, A^	597 ± 66 ^a, A^	27 ± 7 ^d, C^	149 ± 17 ^c, B^	262 ± 31 ^a, A^	197 ± 20 ^b, B^	15 ± 4 ^d, C^
3,9-dihydroxy-megastigma-5 ene	99 ± 12 ^b, A^	118 ± 14 ^ab, A^	117 ± 19 ^ab, A^	128 ± 12 ^a, A^	n.d.	n.d.	n.d.	n.d.	n.d.	n.d.
3-hydroxy-β-ionone	n.d.	n.d.	n.d.	n.d.	n.d.	83 ± 10 ^a, C^	n.d.	n.d.	76 ± 8 ^a, B^	56 ± 8 ^b^
Benzenoids										
benzoic aldehyde	21 ± 7 ^a^	7 ± 3 ^c, B^	14 ± 3 ^b, A^	n.d.	6 ± 3 ^c, B^	29 ± 4 ^a^	22 ± 8 ^ab, A^	1 ± 1 ^d, D^	10 ± 5 ^c^	18 ± 3 ^bc, A^
methyl salicylate	6 ± 4 ^b, D^	9 ± 4 ^b, B^	74 ± 11 ^a, A^	4 ± 3 ^b^	2 ± 2 ^b, C^	18 ± 5 ^a, A^	12 ± 3 ^ab, B^	18 ± 4 ^a, B^	5 ± 2 ^c^	8 ± 4 ^bc, A^
benzyl alcohol	1220 ± 28 ^a, B^	659 ± 79 ^c, B^	720 ± 54 ^c, B^	428 ± 64 ^d, B^	1043 ± 32 ^b, A^	784 ± 47 ^b, C^	626 ± 97 ^c, B^	1148 ± 72 ^a, A^	734 ± 84 ^bc, A^	300 ± 83 ^d, D^
2-phenyl ethanol	490 ± 36 ^c, B^	458 ± 55 ^cd, B^	692 ± 75 ^b, B^	259 ± 44 ^d, C^	1073 ± 227 ^a, A^	362 ± 45 ^c, C^	1139 ± 75 ^a, A^	1088 ± 142 ^a, A^	526 ± 78 ^b, A^	412 ± 44 ^bc, C^
eugenol	n.d.	22 ± 7 ^b, B^	37 ± 9 ^a, B^	13 ± 4 ^b, B^	39 ± 7 ^a, B^	n.d.	n.d.	n.d.	24 ± 13 ^B^	21 ± 7 ^C^
vanillin	n.d.	n.d.	n.d.	n.d.	n.d.	59 ± 5 ^A^	n.d.	n.d.	n.d.	n.d.
methyl vanillate	127 ± 17 ^a, B^	152 ± 29 ^a, B^	35 ± 7 ^b, C^	9 ± 4 ^b, D^	30 ± 14 ^b, A^	162 ± 11 ^a, A^	121 ± 7 ^b, B^	n.d.	20 ± 4 ^c, B^	n.d.
acetovanillone	n.d.	n.d.	n.d.	n.d.	n.d.	n.d.	n.d.	n.d.	n.d.	n.d.
zingerone	48 ± 9 ^a, B^	50 ± 5 ^a, B^	24 ± 9 ^b, B^	11 ± 2 ^b, C^	59 ± 17 ^a, A^	20 ± 8 ^b, C^	134 ± 15 ^a, A^	28 ± 7 ^b, B^	23 ± 5 ^b, B^	30 ± 8 ^b, B^
homovanillic alcohol	81 ± 6 ^b, A^	59 ± 4 ^c, B^	86 ± 11 ^b, A^	43 ± 5 ^d, B^	123 ± 11 ^a A^	18 ± 9 ^b, C^	55 ± 9 ^a, B^	23 ± 3 ^b, D^	13 ± 4 ^b, C^	45 ± 8 ^a, C^
methyl syringate	35 ± 11 ^d, B^	462 ± 65 ^a, B^	238 ± 34 ^b, C^	51 ± 9 ^d, A^	161 ± 22 ^c, A^	585 ± 73 ^b, A^	717 ± 84 ^a, A^	295 ± 42 ^c, BC^	35 ± 8 ^d, B^	6 ± 3 ^d, C^
dihydroconiferyl alcohol	n.d.	n.d.	2 ± 1 ^b, B^	31 ± 13 ^a, B^	4 ± 2 ^b, B^	n.d.	n.d.	n.d.	n.d.	n.d.
Lipid derivatives										
hexanol	112 ± 21 ^c, B^	191 ± 18 ^b, B^	271 ± 15 ^a, A^	89 ± 12 ^c, A^	171 ± 11 ^b, A^	63 ± 9 ^d, C^	242 ± 21 ^a, A^	140 ± 11 ^b, C^	91 ± 9 ^cd, A^	118 ± 25 ^bc, B^
*cis*-3-hexenol	18 ± 5 ^b, B^	20 ± 7 ^b^	53 ± 2 ^a, A^	18 ± 4 ^b, B^	49 ± 6 ^a, A^	8 ± 2 ^b, C^	24 ± 4 ^a^	27 ± 6 ^a, C^	11 ± 3 ^b, C^	14 ± 6 ^b, B^
*trans*-2-hexenol	31 ± 11 ^b, B^	44 ± 6 ^ab, A^	8 ± 7 ^c, C^	37 ± 13 ^b, A^	58 ± 17 ^a, A^	7 ± 4 ^c, D^	8 ± 3 ^c, C^	32 ± 6 ^a, B^	14 ± 3 ^bc, B^	17 ± 5 ^b, C^

^§^ Mean ± standard deviation of three replications of the same sample. n.d. = not detected.

**Table 3:** Content (mean ± SD) ^§^ of higher alcohols (mg/L) of aged red wines. Values with different lowercase letters (^a–e^) differ significantly (Tukey’s test, *p* < 0.05) according to years of aging. Values with different capital letters (^A–D^) differ significantly (Tukey’s test, *p* < 0.05) according to variety.

	**Aglianico**					**Negroamaro**				
	**1** **Year**	**2** **Years**	**3** **Years**	**7** **Years**	**11** **Years**	**1** **Year**	**2** **Years**	**3** **Years**	**7** **Years**	**11** **Years**
Methanol	175 ± 12 ^b, B^	219 ± 14 ^a, B^	101 ± 8 ^d, B^	158 ± 10 ^b, C^	123 ± 14 ^c, B^	132 ± 6 ^d, C^	175 ± 7 ^c, C^	228 ± 11 ^a, A^	220 ± 14 ^a, A^	196 ± 17 ^b, A^
n-propanol	21 ± 3 ^b, B^	19 ± 2 ^bc, B^	14 ± 2 ^c, B^	28 ± 3 ^a, B^	30 ± 5 ^a, AB^	34 ± 15 ^ab, AB^	34 ± 17 ^ab, AB^	11 ± 3 ^c, B^	45 ± 7 ^a, A^	17 ± 5 ^bc, C^
Isobutanol	44 ± 7 ^AB^	42 ± 8 ^C^	39 ± 4 ^B^	52 ± 8 ^AB^	41 ± 3 ^B^	34 ± 5 ^b, C^	32 ± 8 ^b, C^	53 ± 8 ^a, A^	63 ± 6 ^a, A^	54 ± 7 ^a, AB^
Isoamyl alcohol	316 ± 13 ^c, C^	366 ± 19 ^b, A^	265 ± 15 ^d, C^	324 ± 17 ^c, A^	458 ± 23 ^a, A^	437 ± 25 ^a, A^	225 ± 9 ^d, C^	340 ± 21 ^b, B^	166 ± 12 ^e, D^	279 ± 14 ^c, C^
	**Uva di troia**					**Primitivo**				
	**1** **year**	**2** **years**	**3** **years**	**7** **years**	**11** **years**	**1** **year**	**2** **years**	**3** **years**	**7** **years**	**11** **years**
Methanol	117 ± 11 ^cd, C^	260 ± 14 ^a, A^	230 ± 19 ^b, A^	97 ± 11 ^d, D^	121 ± 8 ^c, B^	215 ± 12 ^a, A^	178 ± 13 ^c, C^	96 ± 10 ^d, B^	187 ± 16 ^bc, B^	209 ± 17 ^ab, A^
n-propanol	31 ± 6 ^ab, AB^	38 ± 4 ^a, A^	39 ± 8 ^a, A^	15 ± 3 ^c, C^	26 ± 6 ^b, BC^	40 ± 7 ^b, A^	37 ± 5 ^b, A^	35 ± 4 ^b, A^	55 ± 9 ^a, A^	38 ± 6 ^b, A^
Isobutanol	36 ± 3 ^c, BC^	72 ± 5 ^a, B^	57 ± 9 ^b, A^	31 ± 5 ^c, C^	38 ± 7 ^c, B^	46 ± 5 ^c, A^	95 ± 8 ^a, A^	50 ± 4 ^c, AB^	45 ± 7 ^c, B^	69 ± 8 ^b, A^
Isoamyl alcohol	227 ± 12 ^c, D^	328 ± 17 ^a, B^	271 ± 15 ^b, C^	212 ± 13 ^c, C^	296 ± 14 ^b, C^	383 ± 21 ^a, B^	249 ± 15 ^c, C^	414 ± 23 ^a, A^	254 ± 19 ^c, B^	338 ± 17 ^b, B^

^§^ Mean ± standard deviation of three replications of the same sample.

**Table 4:** Ratios between the mean content of some free forms of glycoconjugates from the enzymatic hydrolysates of grape and aged red wines from 4 varieties.

	**Aglianico**						**Negroamaro**					
	**Grape**	**1** **Year**	**2** **Years**	**3** **Years**	**7** **Years**	**11** **Years**	**Grape**	**1** **Year**	**2** **Years**	**3** **Years**	**7** **Years**	**11** **Years**
***trans*-furan linalool oxide vs.** ***cis*-furan linalool oxide**	**2.1**	**0.92**	**1.00**	**1.41**	**1.15**	**1.10**	**5.2**	**3.53**	**2.53**	**5.14**	**1.61**	**1.36**
***trans*-pyran linalool oxide vs.** ***cis*-pyran linalool oxide**	**0.8–1.5**	**2.33**	**2.57**	**2.60**	**2.00**	**2.19**	**5.9–6.5**	**7.75**	**5.40**	**6.50**	**4.50**	**3.11**
***trans*-8-hydroxy –linalool vs.** ***cis*-8-hydroxy-linalool**	**0.3–0.4**	**0.55**	**0.45**	**0.44**	**0.46**	**0.49**	**0.3**	**0.15**	**0.27**	**0.14**	**0.18**	**0.50**
*trans+cis*-8-hydroxy –linalool vs. *p*-menth-1-ene-7,8-diol	2.5–6.1	2.69	3.21	1.04	0.49	3.0	116–147	14.33	11.40	27.66	0.75	0.71
3-hydroxy-β-damascone vs. 3-oxo-α-ionol	0.1	0.39	0.49	0.09	1.24	0.31	0.34	0.55	0.53	0.65	1.42	0.80
	**Uva di troia**						**Primitivo**					
	**Grape**	**1** **year**	**2** **years**	**3 years**	**7** **years**	**11 years**	**Grape**	**1** **year**	**2** **years**	**3 years**	**7** **years**	**11 years**
***trans*-furan linalool oxide vs.** ***cis*-furan linalool oxide**	**3.0–3.2**	**2.50**	**3.50**	**15.00**	**13.22**	**3.24**	**2.0–3.0**	**2.00**	**1.36**	**3.11**	**1.63**	**1.43**
***trans*-pyran linalool oxide vs.** ***cis*-pyran linalool oxide**	**4.0–5.2**	**4.00**	**4.50**	**9.50**	**7.67**	**9.00**	**2.0–3.0**	**8.50**	**2.22**	**3.23**	**2.55**	**2.63**
***trans*-8-hydroxy –linalool vs.** ***cis*-8-hydroxy-linalool**	**0.8–1.0**	**0.09**	**0.24**	**0.22**	**0.91**	**0.72**	**0.5–0.7**	**0.24**	**0.58**	**0.75**	**0.85**	**0.47**
*trans+cis*-8-hydroxy –linalool vs. *p*-menth-1-ene-7,8-diol	0.5–0.6	2.77	4.50	1.83	0.45	0.16	0.7–1.7	0.60	0.41	3.50	2.06	1.23
3-hydroxy-β-damascone vs. 3-oxo-α-ionol	0.06–0.11	0.60	1.03	0.14	0.23	0.09	0.95–1.3	3.19	1.20	0.43	0.27	6.60

## Data Availability

The original contributions presented in the study are included in the article, further inquiries can be directed to the corresponding author.
